# Mapping freshwater snails in north-western Angola: distribution, identity and molecular diversity of medically important taxa

**DOI:** 10.1186/s13071-017-2395-y

**Published:** 2017-10-10

**Authors:** Fiona Allan, Jose Carlos Sousa-Figueiredo, Aidan M. Emery, Rossely Paulo, Clara Mirante, Alfredo Sebastião, Miguel Brito, David Rollinson

**Affiliations:** 1 Department of Life Sciences, Natural History Museum, Wolfson Wellcome Biomedical Laboratories, Cromwell Road, London, SW7 5BD UK; 2 London Centre for Neglected Tropical Disease Research, London, UK; 3 Centro de Investigação em Saúde de Angola (Health Research Center in Angola), Rua direita do Caxito, Hospital Provincial, Bengo, Angola; 4 Department of Parasitology, Liverpool School of Tropical Medicine, Pembroke Place, L3 5QA, Liverpool, UK; 5 Escola Superior de Tecnologia da Saúde de Lisboa, Lisbon, Portugal

**Keywords:** Angola, Schistosomiasis transmission, *Bulinus globosus*, *Biomphalaria* spp., *Schistosoma haematobium*

## Abstract

**Background:**

This study was designed to determine the distribution and identity of potential intermediate snail hosts of *Schistosoma* spp. in Bengo, Luanda, Kwanza Norte and Malanje Provinces in north-western Angola. This is an area where infection with *Schistosoma haematobium*, causing urogenital schistosomiasis, is common but little is yet known about transmission of the disease. Angola has had a varied past with regard to disease control and is revitalising efforts to combat neglected tropical diseases.

**Methods:**

Snails were sampled from 60 water-contact points. Specimens of the genera *Bulinus*, *Biomphalaria* or *Lymnaea* were screened for trematode infections by inducing cercarial shedding. Snails were initially identified using shell morphology; subsequently a cytochrome *c* oxidase subunit 1 (*cox*1) gene fragment was amplified from a subset of snails from each site, for molecular identification. Cercariae were captured onto FTA cards for molecular analysis. Specimens of *Bulinus angolensis* collected from the original locality of the type specimen have been characterised and comparisons made with snails collected in 1957 held at the Natural History Museum, London, UK.

**Results:**

In total snails of nine genera were identified using morphological characteristics: *Biomphalaria, Bulinus, Gyraulus*, *Lanistes*, *Lentorbis*, *Lymnaea*, *Melanoides*, *Physa* and *Succinea*. Significant for schistosomiasis transmission, was the discovery of *Bulinus globosus*, *B. canescens*, *B. angolensis*, *B. crystallinus* and *Biomphalaria salinarum* in their type-localities and elsewhere. *Bulinus globosus* and *B. angolensis* occurred in two distinct geographical areas. The *cox*1 sequence for *B. globosus* differed markedly from those from specimens of this species collected from other countries. *Bulinus angolensis* is more closely related to *B. globosus* than originally documented and should be included in the *B. africanus* group. *Schistosoma haematobium* cercariae were recovered from *B. globosus* from two locations: Cabungo, Bengo (20 snails) and Calandula, Malanje (5 snails). *Schistosoma haematobium* cercariae were identified as group 1 *cox*1 corresponding to the type common throughout the African mainland.

**Conclusions:**

Various freshwater bodies in north-western Angola harbour potential intermediate snail hosts for urogenital schistosomiasis, highlighting the need to map the rest of the country to identify areas where transmission can occur and where control efforts should be targeted. The molecular phylogeny generated from the samples confirmed that considerable variation exists in *B. globosus*, which is the primary snail host for *S. haematobium* in many regions of Africa.

**Electronic supplementary material:**

The online version of this article (10.1186/s13071-017-2395-y) contains supplementary material, which is available to authorized users.

## Background

Intestinal and urogenital schistosomiasis, caused by the trematode worms *Schistosoma mansoni* and *S. haematobium*, respectively, are endemic in Angola. In 2010, the World Health Organization (WHO) estimated that 60% of Angola’s population were living at significant risk of schistosomiasis infection and required preventive chemotherapy [1]. This prevalence equates to 14.6 million people according to the most recent census [2]. However, these estimates are based on surprisingly few epidemiological surveys and non-systematically gathered data. Due to this lack of up-to-date information, there is an urgent need for further investigations relating to schistosomiasis and other neglected tropical diseases (NTDs) in Angola encompassing epidemiology, transmission studies and operational research.

While much can be done to control morbidity of schistosomiasis using large-scale community or school mass drug administration of praziquantel [3, 4], it is clear that sustainable control and elimination will need a better understanding of transmission and water contact, as highlighted in the WHO roadmap for control of NTDs [5]. Like many other sub-Saharan African countries, Angola is now mapping schistosomiasis, among other NTDs, in humans. However, very little is known about the diversity of freshwater snails responsible for transmission; in fact, the last large-scale survey of freshwater snails was published more than 50 years ago by Wright in 1963 [6].

Looking back further, to the work of Morelet in 1866 and 1868 [7, 8], it is clear that Angola is an important country in the history of medical malacology, from which many important snails were first described. In fact, the type-localities of many species of *Bulinus* and one species of *Biomphalaria* are in Angola: *Bulinus globosus* (Morelet, 1866), River Dande, Bengo Province; *B. angolensis* (Morelet, 1866*)*, district of Duque de Braganza (now Calandula); *B. canescens* (Morelet, 1868), marshes near River Bengo near Quicuxi; *B. crystallinus* (Morelet, 1868); river close to Golungo Alto and *Biomphalaria salinarum* (Morelet, 1868), tributaries to the River Cuije near Malange (see Brown [9]). Snails from Angola have not previously been collected and examined by modern methods of molecular characterization; hence, there is a need to carry out a comprehensive study to learn more about schistosomiasis and the snails responsible for the transmission of this debilitating disease in Angola and their relationships with other African taxa.

This investigation aimed to determine freshwater snail biodiversity and to identify potential intermediate snail hosts of schistosomes in four provinces in north-western Angola. By collecting fresh samples, comparing them to samples collected in the same locations by C. A. Wright nearly 60 years before [6], and subjecting them to modern molecular analysis we attempted to better characterise the species of the genera *Bulinus* and *Biomphalaria* currently found in Angola and to provide new molecular markers for species identification.

## Methods

### Locations

**Fig. 1:**
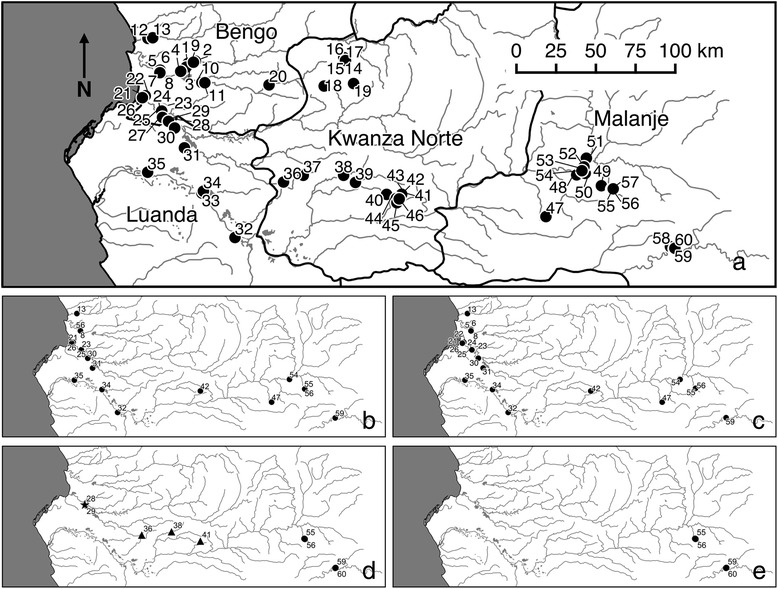
Site location maps. **a** All sites visited in this study. **b**-**e** Sites where the following species were found: **b** *Bulinus gobosus*; **c** *Bulinus truncatus*; **d** *Bulinus canescens* (star), *Bulinus crystallinus* (triangle) and *Bulinus angolensis* (circle); **e** *Biomphalaria salinarum*

Fieldwork was conducted during November and December 2013, by JCSF, AL, AMS, CM and DR. The Dande, Bengo, Kwanza, Lucala and Cuije river basins were visited. Accordingly, a total of four provinces were visited and extensively surveyed: Bengo (25 sites), Luanda (10 sites), Kwanza Norte (12 sites) and Malanje (13 sites), see Fig. 1a for graphical representation and Table 1 for site details.

**Table 1:** Sites visited during field-work

Code	Name	Province	Type	Closest village	Latitude (E)	Longitude (S)	Altitude^a^
1	Mabubas Dam	Bengo	Dam	Mabubas	13.6999	8.5343	70
2	River Dande and marshes	Bengo	Marshes	Sogramo Farm	13.74204	8.52106	65
3	Caxito canal (North)	Bengo	Canal	Caxito	13.68946	8.55546	33
4	Caxito canal (Centre)	Bengo	Canal	Caxito	13.65779	8.57986	19
5	Cabungo stream (type-locality for *Bulinus globosus*)	Bengo	Stream	Cabungo	13.53737	8.57579	22
6	Lake Cabundo	Bengo	Lake	Cabungo	13.53983	8.57282	19
7	Natural dam	Bengo	Dam	Cabungo	13.53779	8.58734	12
8	Irrigation canal	Bengo	Canal	Cabungo	13.53824	8.58765	22
9	River Dande flood plain	Bengo	River	Talelo	13.731	8.52747	69
10	Decomissioned irrigation canal	Bengo	Canal	Icau Centro	13.77906	8.64375	42
11	Lake formed by the River Úcua floodplain	Bengo	Lake	Icau Wando	13.79642	8.6451	49
12	River Lifune	Bengo	River	Libongo	13.46949	8.39041	56
13	Irrigation canal close to Libongo	Bengo	Canal	Libongo	13.49718	8.38692	56
14	River Tanda (downstream of dam)	Bengo	River	Quibaxe	14.59702	8.51995	758
15	River Tanda (upstream of dam)	Bengo	River	Quibaxe	14.59674	8.52083	762
16	Stream Quizende	Bengo	Stream	Quibaxe	14.59362	8.50073	700
17	Merge point between the rivers Tanda and Quizende	Bengo	River	Quibaxe	14.60326	8.51774	742
18	River Calua	Bengo	River	Pango Aluquem	14.4808	8.66434	580
19	River Úcua (1)	Bengo	River	Cacamba	14.64988	8.64988	329
20	River Úcua (2)	Bengo	River	Ucua	14.16441	8.65763	240
21	Lake Panguila (1) (potential type-locality for *Bulinus canescens*)	Bengo	Lake	Porto mangueiras	13.45476	8.71929	0
22	Lake Panguila (2)	Bengo	Lake	Porto mangueiras	13.45443	8.72003	9
23	Canal from Lake Panguila (1)	Bengo	Canal	Burgalheira escola	13.55499	8.79566	18
24	Canal from Lake Panguila (2)	Bengo	Canal	Burgalheira escola	13.55088	8.80342	15
25	Canal from Lake Panguila (3)	Bengo	Canal	Burgalheira escola	13.54862	8.80783	16
26	Bridge over the Lake Panguila	Luanda	Lake	Panguila	13.43812	8.73092	18
27	River Bengo	Luanda	River	Funda	13.55313	8.84427	19
28	Lake Quilunda (1) (potential type-locality for *Bulinus canescens*)	Luanda	Lake	Muculo	13.58681	8.86712	25
29	Lake Quilunda (2) (potential type-locality for *Bulinus canescens*)	Luanda	Lake	Muculo	13.58789	8.86708	23
30	Lake Quilunda (3)	Luanda	Lake	Cadianzala	13.62339	8.90514	20
31	Artificial lagoon on Aurora farm	Luanda	Lagoon	Onga Zanga	13.67903	9.0191	31
32	Pool formed by River Kwanza	Luanda	Pool	Candimba/Muxima	13.96911	9.53437	21
33	Cambemba Lagoon (1)	Luanda	Lagoon	Cacefo	13.77959	9.27866	18
34	Cambemba Lagoon (2)	Luanda	Lagoon	Cacefo	13.78779	9.26822	16
35	Canal from the River Kwanza	Luanda	Canal	Caquila	13.468	9.1594	4
36	Pool near Aldeia Nova (1) (type-locality for *Bulinus crystallinus*)	Kwanza Norte	Pool	Aldeia Nova	14.24768	9.21573	201
37	Pool near Aldeia Nova (2)	Kwanza Norte	Pool	Aldeia Nova	14.36089	9.1771	239
38	Unknown stream near Golungo Alto	Kwanza Norte	River	Golungo Alto	14.59253	9.17718	308
39	River Luinha	Kwanza Norte	River	Luinha	14.66055	9.21806	333
40	River Lussué	Kwanza Norte	River	Lussue	14.83917	9.2864	591
41	River Muembege (1)	Kwanza Norte	River	N’Dalatando	14.92749	9.28563	793
42	River Muembege (2)	Kwanza Norte	River	N’Dalatando	14.92782	9.28517	804
43	River Muembege (3)	Kwanza Norte	River	N’Dalatando	14.92582	9.28576	801
44	River Cangulungo (potential type-locality for *Bulinus crystallinus*)	Kwanza Norte	Stream	N’Dalatando	14.89462	9.31063	759
45	River Muembege (4)	Kwanza Norte	River	N’Dalatando	14.89815	9.33458	725
46	River Muembege (5)	Kwanza Norte	River	N’Dalatando	14.91162	9.31264	765
47	River Cambota	Kwanza Norte	River	Cacuso	15.75334	9.4143	1045
48	River Quimona	Malanje	River	Soqueco	15.92973	9.1746	971
49	River Memba	Malanje	River	Bingwe	16.07151	9.23689	1079
50	Unknown stream near Carlanga	Malanje	Stream	Carlanga	15.97861	9.16565	1026
51	River Quialeva	Malanje	River	Calandula	15.98556	9.0786	1084
52	River Lucala	Malanje	River	Vulabongo	15.97136	9.12839	959
53	River Sende (1)	Malanje	River	Capoza	15.96031	9.15152	986
54	River Sende (2)	Malanje	River	Capoza	15.95987	9.1505	983
55	River Camahonjo (1)	Malanje	River	Mangumbala	16.13156	9.25788	1092
56	Source of the River Cota (type-locality for *Bulinus angolenses*)	Malanje	River	Mangumbala	16.13693	9.26186	1083
57	River Camahonjo (2) Source	Malanje	River	Leco Segundo	16.13938	9.25468	1111
58	River Calulo	Malanje	River	Quissol	16.46778	9.58705	1038
59	River Quastimbala (1) (type-locality for *Biomphalaria salinarum*)	Malanje	River	Catunga	16.48999	9.59848	1047
60	River Quastimbala (2) (type-locality for *Biomphalaria salinarum*)	Malanje	River	Catunga	16.49321	9.59602	1051

^a^Metres above sea level

### Malacology procedures

Water bodies were examined for freshwater snails using a standard protocol [9]. Information was recorded on pre-designed forms and included: general locality information (water body name, type, GPS coordinates, nearby village); water properties data (temperature, pH, salinity, dissolved solids, flow rate, water level, conductivity, depth); collector information (number of collectors, time/length of time of collection), ecological data (substrate, all species of snail present, vegetation, animal contact, human contact), and snail data (snails species, abundance, number infected).

Snails were collected using large scoops, hand sieves and forceps for approximately 22 min per site usually with 3–4 persons. Snails were carefully picked out with forceps taking care not to damage the shells. The collection at each site was separated into genera and placed in screw cap pots with clean mineral water before securing in a cooler with ice packs (when available) for transportation. Photographs were taken of each site visited. Upon return to Centro de Ivestigação em Saúde de Angola (CISA) (Angolan Health Research Center, in Caxito) *Bulinus* and *Biomphalaria* specimens were placed in fresh water and checked for the shedding of cercariae by exposing them to a lamp or sunlight, any emerging cercariae were examined under the microscope and identified by morphology. Snails were screened, individually, for a period of 24 h and then fixed in ethanol. Individual cercariae in 4 μl of water, collected by pipetting, were placed onto Whatman FTA Classic cards (GE Healthcare Life Sciences, Amersham, UK) for future molecular analyses [10]. Snails were relaxed by briefly placing in a -20 °C freezer and then fixed in ethanol (95%). Screw top 25 ml universal tubes were labelled both internally using pencil and parchment paper and externally with permanent marker.

### Molecular characterization studies

#### Sample preparation and DNA extraction

The snail samples selected for the molecular analyses represented individuals from each collection site. Examples of all species of *Bulinus* and *Biomphalaria* found in this collection trip were included. Additionally, a single sample from C. A. Wright’s 1957 collection held at the Natural History Museum was included in these analyses. All specimens were stored in 95% ethanol in the field and the number of snails recounted, identified by morphological characters and re-spirited (absolute ethanol) on arrival at the Natural History Museum, London (NHM) for incorporation in to the Schistosomiasis Collection at the Natural History Museum (SCAN) [11]. Photographic images were taken of the snail shells prior to DNA extraction. Specimens were placed in TE buffer (10 mM Tris, 0.1 mM EDTA) pH 7.4 for 1 h in order to remove any remaining alcohol from within the tissue, which might interfere with subsequent extraction techniques. Total genomic DNA was isolated from head-foot snail tissue using the DNeasy Blood and Tissue kit (Qiagen, Crawley, UK) according to the manufacturer’s instructions except that the volume of buffers was doubled. DNA was eluted into 200 μl sterile water.

#### Amplification of *cox*1 fragments of snail DNA

A polymerase chain reaction (PCR) amplification of a partial cytochrome *c* oxidase subunit 1 (*cox*1) sequence was performed using primers LCO1490 (5′-GGT CAA CAA ATC ATA AAG ATA TTG G-3′ forward) and HCO2198 (5′-TAA ACT TCA GGG TGA CCA AAA AAT CA-3′ reverse) [12]. PCR investigations and sequencing conditions were chosen as previously outlined [13]. A second set of primers was required for the PCR and sequencing of *B. globosus* and *B. angolensis* specimens; these were designed using whole mitochondrial genome data for these species (novel): BulAng61F (5′-GTA TGA TGC GGC CTG GTA GG-3′) and BulAng895R (5′-AAG CCC GAG TAT CCA CAT CT-3′). The PCR conditions were as outlined by [13] except an annealing temperature of 60 °C instead of 40 °C was used. Sequencing was performed on an Applied Biosystems 3730XL analyser (Life Technologies, UK).

#### Extraction and amplification of *cox*1 and ITS2 fragments of *Schistosoma haematobium*

Punches (2.0 mm) were taken from the centre of each cercarial spot on FTA cards and pH elution performed to remove the DNA from the card matrix [14]. A 1500 bp fragment was amplified from 3 μl of genomic DNA using the Cox1_Schist_5′ and Cox1_Schist_3′ [15] primers and illustra™ puReTaq Ready-To-Go PCR Beads (GE Healthcare Life Sciences, UK). The following cycling conditions were used: 95 °C for 1 min, 40 cycles of 95 °C for 30 s, 40 °C for 30 s and 72 °C for 2 min, with a 7 min extension of 72 °C. In addition, ITS2 was amplified as it would identify schistosome hybrids, the PCR used ITTS1 and ITTS2 primers [15] with the above conditions except the annealing temperature of 56 °C instead of 40 °C. PCR products were cleaned using the QIAquick PCR purification kit (Qiagen, UK). Samples were sequenced in both orientations on an Applied Biosystems 3730XL analyser running BigDye v3.1 sequencing chemistry.

#### Phylogenetic analysis of sequence data

The electropherograms produced were checked and *cox*1 sequences edited using Geneious, version 6.1.8 (http://www.geneious.com [16]). Sequences were compared to database entries by performing BLAST searches via the National Center for Biotechnology Information against GenBank and EMBL sequence databases; and aligned with reference material [13] using Geneious version 5.6. The *cox*1 data for all taxa were analysed solely as nucleotides and phylograms were produced from the alignments using PhyML with automatic model selection by SMS [17, 18] using the Akaike Information Criterion. Branch support was estimated using 1000 bootstrap replications. Additionally, *B. truncatus* (Niger [13]) was used as an outgroup for the *Bulinus forskalii* species group, and *B. forskalii* (Niger [13]) was used for the *Bulinus africanus* species group. Different outgroups were used for the different analyses based on suitability (geographical and sister taxa). DNA sequences have been submitted to the European Nucleotide Archive with accession numbers LT671915–LT671982.

## Results

### Locations

A total of 60 sites were visited and included rivers, streams and canals. Sites on the Bengo, Kwanza and Cuije River Basins displayed the highest mean snail biodiversity, with more than 3 genera identified (Table 2). In total, 73% of sites had a mud substrate (usually associated with roots of aquatic and peripheral vegetation), with little domestic animal water contact observed (of the 60 sites 3% had cows, 8% goats, 13% pigs, 7% dogs, 3% horses and 7% had chickens). Wildlife was present in some sites: in 18% of the sites aquatic reptiles were observed or believed to be present, in 3% of the sites hippopotamus were observed or believed to be present, and in 27% of the sites aquatic birds were observed. Finally, the most prevalent vegetation identified in all 60 sites was grass (57%), macrophyte plants (52%), lilies (37%), rushes (35%) and water hyacinths (13%), in descending order. The water chemistry and other factors did not significantly impact on the presence or absence of snail species (Table 2; Additional file 1: Table S1).

**Table 2:** Summary of water chemistry parameters and biodiversity of each river basin

River basin	Dande/Úcua	Bengo	Kwanza	Lucala	Cuije
Number of sites	20	11	4	22	3
Altitude (m)	232 (12–762)	17 (0–31)	14 (4–21)	220 (201–1111)	1045 (1038–1051)
Temperature (°C)	28.3 (25.4–32.8)	30.4 (27.8–33.1)	32.7 (30.9–33.6)	31.5 (22.5–32.1)	24.2 (22.1–27.2)
TDS (ppm)	205 (42–736)	398 (138–830)	69 (59–80)	119 (26–612)	204 (156–276)
pH	7.75 (6.96–8.79)	7.71 (6.88–8.70)	7.02 (6.54–7.82)	9.26 (6.85–9.60)	7.76 (7.07–8.31)
Conductivity (m/s)	245 (59–795)	562 (194–1171)	96 (83–112)	159 (65–860)	288 (220–397)
Salinity (ppm)	125 (33–389)	276 (95–581)	52 (46–58)	84 (35–415)	139 (106–190)
Average biodiversity (no. of genera)	1.35 (0–4)	3.27 (2–5)	3.25 (3–4)	1.5 (0–2)	3.3 (1–5)

*Note*: Data are given as mean (range)

### Malacology

In total 1265 snail samples were accessioned in to SCAN. According to morphological evaluations, species of a total nine snail genera were identified: *Biomphalaria*, *Bulinus*, *Gyraulus*, *Lanistes*, *Lentorbis*, *Lymnaea*, *Melanoides*, *Physa* and *Succinea*..Of most significance for schistosomiasis transmission, was finding of *Bulinus globosus* (105 specimens), *Bulinus canescens* (3 specimens), *Bulinus angolensis* (8 specimens), *Bulinus crystallinus* (> 200 specimens) and *Biomphalaria salinarum* (23 specimens) in what are believed to be their type-localities and other habitats (Fig. 1).

Subsets of the snails (representatives of every site and species) were used for molecular analyses (*n* = 133). *Bulinus globosus* and *B. forskalii* were the most abundant snail species and covered the greatest range of sites; 403 specimens in 21 sites and 390 specimens in 12 sites, respectively. Other species were: *Lymnaea natalensis*, *Melanoides tuberculata*, *Lanistes ovum*, *Gyraulus costulatus*, *Lentorbis benguelensis*, *Succinea* sp. and *Physa acuta.*

### Analysis of sequence data

**Fig. 2:**
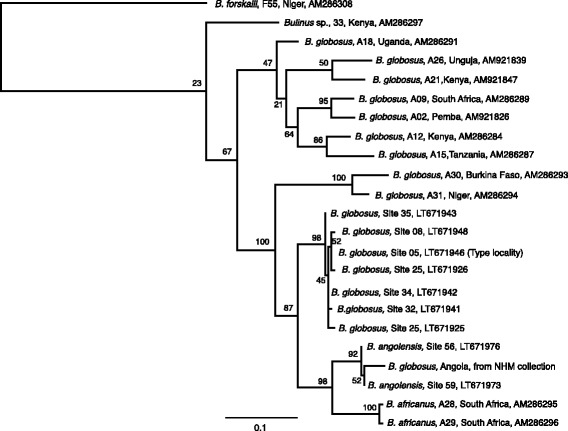
Maximum Likelihood tree for *Bulinus globosus* and *B. angolensis* based on *cox*1 sequences. Maximum likelihood tree of a 613 bp fragment of the cytochrome *c* oxidase subunit 1 (*cox*1) gene for *B. globosus* and *B. angolensis* collected in this study, with additional previously published sequences [13]. *B. forskalii* was selected as the outgroup. Numbers at the nodes indicate bootstrap support (1000 pseudoreplications). The scale-bar indicates substitutions per nucleotide site

**Fig. 3:**
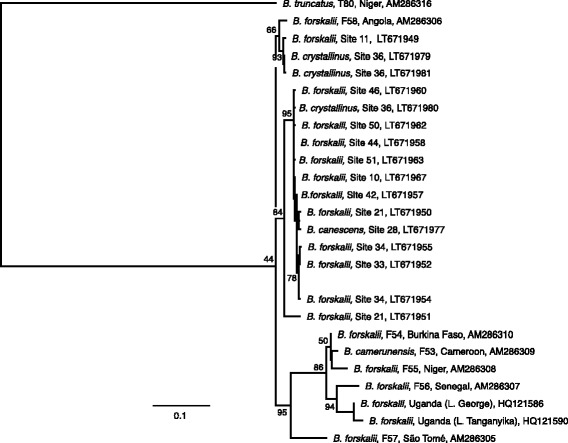
Maximum Likelihood tree for *Bulinus forskalii, B. crystallinus* and *B. canescens* based on *cox*1 sequences. Maximum likelihood tree of a 590 bp fragment of the cytochrome *c* oxidase subunit 1 (*cox*1) gene for *Bulinus forskalii, B. crystallinus* and *B. canescens* collected in this study, with additional previously published sequences. *B. truncatus* was selected as the outgroup. Numbers at the nodes indicate bootstrap support (1000 replications). The scale-bar indicates substitutions per nucleotide site

The mitochondrial *cox*1 gene sequence generated for all the *Bulinus* species in this study was found to be variable, in agreement with previous work [13]. Initially the universal “Folmer” primers, effective across a wide range of taxa, were used that cover approximately the first 600 bp of *cox*1; however for *B. globosus* and *B. angolensis* these standard primers did not amplify the target region. By examining whole mitochondrial DNA sequence (unpublished) new primers were designed for these species, which allowed the amplification of a comparable region of DNA sequence. Phylogenetic analysis of the sequence data for snails identified as *B. globosus* and *B. angolensis* revealed that the *B. globosus* snails collected for this study formed a monophyletic group distinct from *B. globosus* found elsewhere (Fig. 2). Additionally, the snails identified as *B. angolensis* formed another distinct clade with the specimen from Wright’s 1957 study, previously identified as *B. globosus.* Snails from Angola identified as *B. forskalii* were not monophyletic with respect to *B. canescens* or *B. crystallinus*, whereas *B. forskalii* (and *B. camerunensis*) from elsewhere formed a separate clade (Fig. 3). Phylogenetic analysis of *Biomphalaria salinarum* suggested a close affinity with *Biomphalaria pfeifferi* (not shown); DNA sequence was highly similar to *B. pfeifferi* for the *cox*1 barcoding region (up to 99% similarity).

### Parasitology

Of the *Bulinus* spp. and *Biomphalaria* spp. specimens collected, only *Bulinus globosus* (25 in total) was found to be shedding *Schistosoma* cercariae. These were found in 2 sites: Site 5 [20 out of 105 snails shedding (19% prevalence)] and site 60, the source of the River Cata [5 out of 68 (0.07% prevalence)]. The *cox*1 and ITS2 sequences of the cercariae sampled corresponded to haplotype H1 of *S. haematobium*, by far the most widespread across Africa [19]. Apart from human parasites, *B. globosus* specimens were also found to be shedding *Trichobilharzia*, echinostome, amphistome and strigeid cercariae. *Bulinus crystallinus* specimens were found to be shedding cercariae of *Trichobilharzia* and echinostomes. The non-schistosome cercariae were identified using morphology and swimming characteristics.

## Discussion

Angola is a particularly rich area for type-localities of both *Bulinus* and *Biomphalaria* species and it is of interest that similar diversity of snails still exists in locations sampled 56 years previously. The fact that the survey was conducted in November/December had its advantages and disadvantages. Importantly, C. A. Wright visited these same areas in November/December of 1957 [6], giving concordance in sampling season between the two studies. However, the survey was conducted at the start of the rainy season, when many of the rivers and tributaries were running low or even dry (namely in the Úcua River basin). Nevertheless, the level of snail biodiversity in some sites was high, as well as the abundance. For example, in the Caxito canal (sites 3 and 4), the number of *Melanoides tuberculata* and *Physa acuta* was large and may in part account for the absence of *Bulinus* spp.

The 37 recognised species of *Bulinus* have been divided for convenience into fourgroups: the *B. africanus* group, the *B. reticulatus* group, the *B. forskalli* group and the *B. truncatus/tropicus* complex [9], while others studies have argued for subdivision of the genus into three genera [20]. The lack of clear morphological characters for species identification provided the impetus to search for molecular solutions and *cox*1 barcoding was shown to provide good species discrimination and agreed for the most part with the taxonomy based on morphological criteria [9]. The dataset generated here can be added to and compared with the earlier *cox*1 sequences considered by Kane et al. [13].

One of the most striking findings is the close association observed between *B. globosus* and *B. angolensis*. The latter species had previously been placed in a different species groups: *B. globosus* within the *B. africanus* group while *B. angolensis* had been included in the *B. tropicus/truncatus* complex. Brown [9] made the point that treatment of this species is unclear partly because the chromosome number and molecular properties were unknown. Wright [6] noted that he did not have sufficient material to assess properly the relationships of this species or to fully describe the range of variation. Our own material has so far been limited but based on current data it is safe to consider *B. angolensis* as a member of the *B. africanus* group and not a member of the *B. tropicus/truncatus* complex.

Another observation that deserves further, detailed studies is the relationship of the samples included as *B. globosus.* Kane et al. [13] drew attention to the fact that there was a clear division between samples originating from East and West Africa. *Bulinus globosus* has almost a pan-African distribution. The trees generated here on *cox*1 sequence data suggest that *B. globosus* from Angola are quite distinct from other geographical areas. This observation might cause a taxonomic dilemma as the samples analysed here are in fact from the type-locality and hence represent the “true” *B. globosus*. It is clear that this snail species, which acts as an important host for *S. haematobium* and other schistosome species throughout its range, needs a more thorough investigation. While changes in nomenclature might eventually be warranted we suggest that further sampling is required and more comprehensive molecular data are needed before such changes are made.

Additionally, a single sample from C. A. Wright’s 1957 collection held at the Natural History Museum as part of SCAN was included in these analyses and can be seen to be most similar to *B. angolensis* from the 2013 collection. The *cox*1 sequence gained from this snail is particularly interesting as the specimen had been held in industrial methylated spirit for the past 60 years; samples held in this way are generally fragmented as the DNA can be damaged by this storage medium. Wright’s original identification on morphological grounds alone was that this specimen was best considered as *B. globosus* but the molecular data strongly supports the grouping with *B. angolensis*.

Other specimens collected at type-localities were from the *B. forskalii* species group. Again, analysis of the *cox*1 regions shows that the samples from Angola are diverse and not necessarily falling into the groups that were previously expected. It is clear that there are at least two different groupings (Fig. 3); first “*B. forskalii*” types with the inclusion of *B. canescens* potentially as a subgroup of *B. forskalii*, and the *B. crystallinus* types which are distinct from the main clade. It is of interest to note here that Brown [9] discussed the lumping of these species and thought that there may be up to 12 separate species within Angola; these points were also made by Jones et al. [20] and Kane et al. [13]. Species assignment based on locality and morphology looks somewhat arbitrary when compared with phylogenetic analysis using sequence data, especially for the *B. forskalii* group. While *cox*1 barcoding has been used extensively for snail vectors (e.g. [13]), it is clear that further investigation at the molecular level is needed to elucidate and identify and relationship of species within this species group prior to any changes in nomenclature.

*Biomphalaria* species were collected from only a couple of locations in this study, ultimately only two specimens had unique *cox*1 sequence and therefore were included in this analysis. From the analysis *B. salinarum* is found at two sites and is most closely related to *B. pfeifferi.*

With reference to the schistosome species found in this study, to our knowledge this is the first time that *S. haematobium* has been genetically typed from Angola. *Schistosoma haematobium* cercariae recovered from *B. globosus* showed to be standard H1 *cox*1 haplotype and the three samples examined had a low diversity (only 3 single nucleotide changes). This is what we would expect from the African mainland from previous studies [19]. No other animal schistosomes were encountered, but a range of species of cercariae were produced by a number of species of snail; this is usual in water-contact sites in use by humans, animals (wild and domestic) and birds. Interestingly, *Biomphalaria* species were not common suggesting that intestinal schistosomiasis caused by *S. mansoni* is unlikely to be a major public health problem in the region [21].

As schistosomiasis control programmes gather pace it is important that malacological studies are carried out to help guide mapping of disease transmission and the planning of control interventions. Focus is moving towards the elimination of schistosomiasis [4, 5] and a recent analysis has shown the value of interventions that control snail populations [22]. The increase in data collection from sites, with geo-referencing, and with molecular techniques gives a more detailed view on transmission and where to target control efforts. This study has initiated mapping of snail distributions in four provinces of Angola, important site localities have been geo-referenced and photographic records have been made at each of the collection points. There is now a need to extend the survey into other regions of Angola.

## Conclusions

The findings reported here provide new insights into the molecular diversity within and between the *Bulinus* species examined. The samples of medically important snails collected and identified by the barcoding approach will be subjected to further more in depth molecular sequencing to help elucidate the relationships with *Bulinus* and *Biomphalaria* species from other African regions.

## Additional file

Additional file 1: Table S1. Details of snails at each site. (XLS 48 kb)
